# Therapeutic Peptides in Aesthetic, Metabolic and Endocrine Conditions: Effects, Safety, Clinical Applications, and Future Perspectives

**DOI:** 10.3390/ijms27093890

**Published:** 2026-04-27

**Authors:** Guilherme Renke, Lucas Chinellato

**Affiliations:** 1 Nutrindo Ideais Performance and Nutrition Research Center, Rio de Janeiro 22411-040, Brazil; 2 Instituto Lucas Chinellato, Campinas 13092-133, Brazil; administracao@institutochinellato.com

**Keywords:** therapeutic peptides, GLP-1 analogues, tesamorelin, CJC-1295, ipamorelin, BPC-157, thymosin, GHK-Cu, PT-141, elamipretide

## Abstract

Therapeutic peptides are short chains of amino acids used to treat metabolic and endocrine conditions such as obesity and type 2 diabetes. While several peptide drugs have undergone rigorous approval processes that evaluate both safety and efficacy, novel, unapproved compounds have emerged and are rapidly expanding into preventive medicine and performance enhancement. Our objective is to present the effects, clinical applications, safety profiles, and regulatory status of prominent peptides used to treat several conditions. We reviewed 106 articles, prioritizing systematic reviews, meta-analyses, and randomized controlled trials in the PubMed, ScienceDirect, and SciELO databases. Our results suggest that therapeutic peptides are a promising tool for treating type 2 diabetes and obesity, for skin rejuvenation, and as hormone analogs for specific diseases and conditions. Although these are strategic and innovative options that can improve health, performance, and longevity, further studies are needed before most new peptides can be used safely in humans.

## 1. Introduction

Peptide therapy has become prominent in metabolic diseases, obesity, sports, aesthetics, and longevity in recent years. These compounds are short chains of amino acids (smaller than proteins) that function as biological signaling molecules, modulating specific responses in target tissues, hormone levels, and even repair processes in the body [1,2]. Historically, various peptides have been used in medicine; for example, insulin has been used since 1922, and growth hormone (GH) and its analogs since the mid-20th century [3]. However, in recent decades, there has been an increase in the development and use of synthetic peptides to treat diseases, improve performance, and enhance health [4]. While peptides were once restricted to laboratory and research settings, peptides such as GH-releasing peptides emerged in the 1990s and were rapidly adopted by bodybuilders for experimental use without regulatory approval [5]. Today, with advances in biotechnology, new peptide samples are being tested, with potential benefits ranging from weight loss to skin or tissue regeneration and longevity [6,7].

In the current context of improving metabolic diseases, physical performance, body composition change, dermatology, and injury recovery, several synthetic peptides have been explored and tested in clinical studies. On the other hand, unfortunately, reports from online forums and wear clinics describe “stacks” (combinations) of peptides and other substances to maximize muscle gain, fat burning, injury recovery, and even anti-aging effects [8]. At the same time, some approved peptides are used in cosmetic creams, ranging from collagen-stimulating tripeptides to clinically approved weight-loss injections, such as Glucagon-like peptide-1 (GLP-1) analogs [8,9]. This exponential growth raises regulatory concerns: many peptides remain in a legal grey area, with products of uncertain origin, limited robust clinical studies, and exceptions in competitive sports (doping) [10]. Several peptides are derived from endogenous hormones. For example, GLP-1 (an intestinal incretin hormone) gave rise to antidiabetic drugs; thymus peptides inspired immunomodulatory agents; fragments of growth hormone and ghrelin led to GH secretagogues; and artificial peptides, such as Body Protection Compound (BPC)-157, were discovered in academic research (at the University of Zagreb in the 1990s) [11]. In the last two decades, peptide engineering and medicinal chemistry have enabled the development of more effective and potent molecules, thereby catalyzing the emergence of new applications across diverse clinical conditions [1,2,4,8].

**Figure 1:**
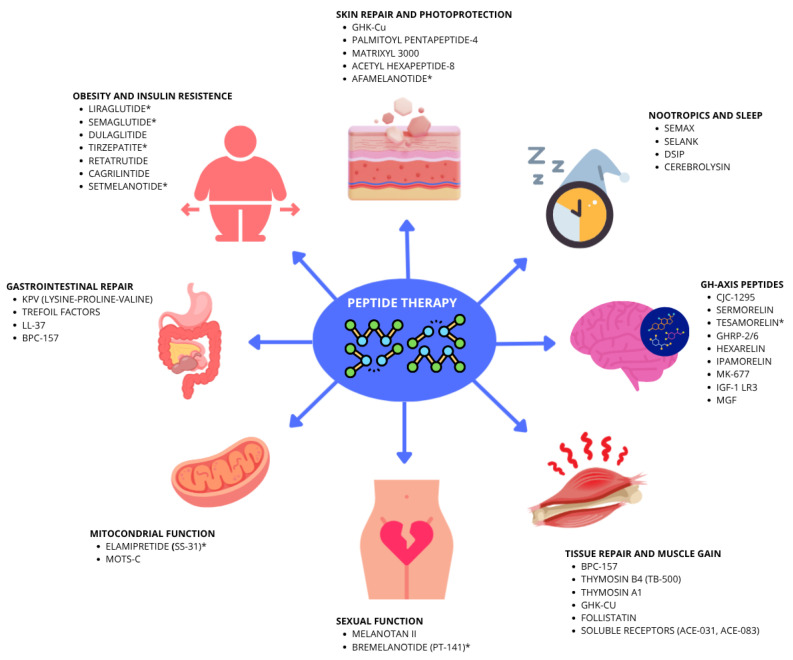
Effects and applications of therapeutic peptides in different clinical conditions. Abbreviation: ACE: Myostatin inhibitory peptide; BPC-157: Body Protection Compound 157; CJC-1295: drug affinity complex: growth hormone-releasing factor; DSIP: delta sleep-inducing peptide; GH: growth hormone; GHK-Cu: Copper glycylhistidyllysine; GHRP: growth hormone-releasing peptides; IGF-1: insulin growth factor 1; KPV: Lys-Pro-Val tripeptide; LL-37: cathelicidin antimicrobial peptide; MK-677: Ibutamoren; MOTS-c: mitochondrial open reading frame of the 12S rRNA-c. * FDA-approved peptides. Figure created with Canvas (Version 2026.01).

However, healthcare professionals should be aware of the Food and Drug Administration (FDA) approval status of these new peptides and their clinical applications (Figure 1). Anticipating the “future” of peptides enables physicians to implement evidence-based interventions, maximize benefits (including pathologies, performance, aesthetics, longevity, and regeneration), and minimize risks associated with clandestine or unsupervised use [12,13].

Thus, the objective of this narrative review is to present the effects, clinical applications, safety profiles, and regulatory status of peptides that are already approved and promising for the treatment of various health conditions, including type 2 diabetes (T2DM), obesity, skin rejuvenation, aging, hormone analogs, and other specific conditions.

### 1.1. Physiology and Development of Peptides

#### 1.1.1. Synthesis and Structure

Peptides are molecules formed by the binding of amino acids in sequence (Table 1). In the body, many hormones and local signaling are peptides (e.g., insulin, GH, growth factors) [7]. Synthetic peptides, on the other hand, can be produced by chemical synthesis (solid-phase technique) or by biotechnological methods (recombinant deoxyribonucleic acid (DNA) or natural extracts). Small structural modifications—such as acylation, cyclization, and the addition of chemical groups—are often employed to increase the stability of these peptides in the body, conferring resistance to enzymatic degradation or directing their action to certain receptors [14]. In general, peptides have a flexible structure and high binding specificity for target proteins (membrane receptors, enzymes, or intracellular signaling factors) [1,2,6,7,14].

#### 1.1.2. Cell Signaling Mechanisms

Synthetic peptide drugs largely originate from endogenous hormones and neuropeptides, but they are usually similar rather than identical in structure or function. Chemists modify natural sequences to improve stability, selectivity, or duration of action, or to create antagonists rather than agonists. They normally act by activating specific receptors on the cell surface. Many act as agonists at G protein-coupled or tyrosine kinase receptors, triggering signaling cascades that modulate enzyme and gene activity [6]. For example, incretinomimetic peptides such as GLP-1 agonists and Gastric inhibitory polypeptide (GIP) bind to G protein-coupled receptors (GPCRs) in the pancreas, increase cAMP levels, and promote glucose-dependent insulin release [12]. GH axis peptides (GHRPs) activate the ghrelin receptor (GHS-R1a), elevating intracellular calcium and stimulating GH secretion by somatotrophic cells [15]. Peptides such as Insulin-like Growth Factor 1 (IGF-1) or derivatives activate tyrosine kinase receptors (IGF-1R) and trigger anabolic pathways (PI3K-Akt-mTOR) [5]. Many peptides exert pleiotropic effects: in addition to their primary effect (such as hormone release), they can modulate inflammation, angiogenesis, or apoptosis via secondary pathways [16].

#### 1.1.3. Routes of Administration and Absorption

Due to their protein nature, most peptides are not orally stable—digestive enzymes degrade them into smaller amino acids. Thus, parenteral administration is predominant. Subcutaneous (SC) injections are the most common route (e.g., weekly SC semaglutide; BPC-157 SC daily) [13]. Some are administered intramuscularly or intravenously (e.g., Cerebrolysin intravenous (IV); delta-sleep-inducing peptide (DSIP) intramuscular (IM). Certain peptides have been formulated for intranasal administration, leveraging the rich vascularization and direct contact with the olfactory cerebrospinal fluid to reach the central nervous system (e.g., Semax and Selank in nasal drops) [17,18,19,20,21]. Peptides are available in topical/transdermal formulations for local action (e.g., GHK-Cu in dermatologic creams). Recently, advances have enabled oral administration of some peptides, including MK-677 (an oral GH secretagogue) and oral semaglutide (a gastric-absorbent formulation) [5,15]. In general, however, the oral bioavailability of peptides is low, and strategies such as encapsulation, lipid conjugation, or intestinal permeation technologies are necessary to make it feasible [22,23]

#### 1.1.4. Metabolism and Half-Life

Injected peptides rapidly enter the circulation and peripheral tissues, and are typically metabolized by endogenous peptidases and proteases (e.g., serum enzymes such as Dipeptidyl peptidase 4 degrade incretins; aminopeptidases degrade neuroactive peptides in the cerebrospinal fluid) [7,24]. Thus, many have a short half-life (minutes to hours). To get around this, long-acting versions have been developed—for example, CJC-1295, a Growth hormone-releasing hormone (GHRH) analog, has been conjugated to a group that binds to albumin, extending its half-life to ~1 week; peptides such as liraglutide and semaglutide have gained coupled fatty acids that delay their renal elimination and proteolysis [16,25]. Small endogenous peptides, on the other hand, can have extremely short half-lives (DSIP has a half-life of minutes and is rapidly eliminated) [26]. Final excretion usually occurs by the kidneys (free amino acids or small fragments are filtered out). Important: due to protein degradation, peptides accumulate only minimally in the body; on the other hand, they require frequent or continuous administration for sustained effects, unless their structure is optimized for prolonged duration [15,27].

Additionally, the development of therapeutic peptides involves balancing the high specificity of action (driven by molecular design) with adequate pharmacokinetic properties (sufficient half-life and appropriate formulation). Below, we will present a classification of the main peptides by mechanism of action, detailing for each category: mechanism, clinical/experimental indications, typical dosage, adverse effects, scientific evidence, and regulatory status (including approval by regulatory agencies such as FDA, European Medicines Agency (EMA), and World Anti-Doping Agency (WADA) status) and Brazil’s Health Regulatory Agency (ANVISA) [7,14].

## 2. Results

### 2.1. Classification of Peptides by Mechanism of Action

#### 2.1.1. Metabolic and Incretin Peptides (GLP-1, GIP, Amylin)

This category encompasses peptides that modulate energy metabolism, mainly via incretin and satiety effects. The main ones are GLP-1 agonists, double/triple agonists (GLP-1 + GIP ± glucagon), and amylin analogs, which act synergistically to regulate appetite, insulin secretion, and body weight [28].

- **GLP-1 receptor agonists (GLP-1 RAs):** These mimetics of the incretin hormone GLP-1 amplify glucose-dependent insulin secretion, inhibit glucagon secretion, and delay gastric emptying. The result is increased satiety and better glycemic control [12]. These peptides (e.g., liraglutide, semaglutide, dulaglutide, exenatide) act on receptors in the pancreas and on the hypothalamus and vagus nerve, suppressing appetite [7]. In addition to metabolic effects, they have systemic benefits, including reduced inflammation and oxidative stress, improved endothelial function, renal protection, and possibly neuroprotection [29].
- **Double/triple agonists (GLP-1 + GIP ± glucagon):** They combine multiple incretin and hormonal effects. The main example is tirzepatide (a dual GIP/GLP-1 agonist): it activates both the GIP and GLP-1 receptors, which confer synergism—GIP enhances the action of GLP-1 in the pancreas and the central nervous system, leading to greater appetite reduction and improved insulinemic sensitivity [28]. Emerging tri-hormonal agonists, such as retatrutide, also activate the glucagon receptor, increasing energy expenditure and hepatic lipolysis [25]. These combinations result in even greater weight loss than isolated agonists. Thus, retatrutide can reduce >20% of body weight in 48 weeks—an unprecedented level among anti-obesity pharmacological therapies [30]
- **Amylin analogues:** Amylin is a hormone co-secreted with insulin by pancreatic beta cells, which contributes to glycemic and satiety control (delays gastric emptying, suppresses postprandial glucagon, and promotes central satiety). The analogue pramlintide has been approved for type 1 and T2DM, but its use for weight loss is limited [4]. New analogues, such as cagrilintide (a long-acting amylin analogue), have been studied for the treatment of obesity. When cagrilintide is combined with semaglutide, an additive effect on weight loss is observed (~15–18% weight reduction in phase 2 studies), suggesting that the coactivation of distinct satiety pathways (GLP-1R and amylin receptor) produces extra benefit [31].
- **Indications and Clinical Evidence:** Incretin peptides have revolutionized the management of obesity and T2DM. Robust clinical trials document its effectiveness. In the STEP study, semaglutide 2.4 mg weekly was associated with a mean weight loss of ~15% (up to 17% at 68 weeks) [32]. In the SELECT study with obese non-diabetic patients, semaglutide reduced the incidence of major cardiovascular events (infarction, stroke) by ~20% at 5 years [33]. It has also demonstrated renoprotective effects in patients with T2DM and nephropathy [34]. Emerging evidence of cognitive benefit: Alzheimer’s disease models treated with semaglutide showed improved memory and reduced neuroinflammation [13]. Semaglutide is approved globally for T2DM and obesity, due to the high level of evidence [14].
- **Tirzepatide (dual GIP/GLP-1):** In T2DM, the SURPASS-2 study showed that tirzepatide reduced glycated hemoglobin (HbA1c) by ~2.3% and weight by 11 kg at 72 weeks [35]. In patients with obesity without T2DM, the SURMOUNT-1 study reported an average weight loss of 17.8% at 72 weeks [36]. In addition, improvements in metabolic parameters, such as lipid profile, liver fat (steatosis), and inflammatory markers (CRP), have been observed with tirzepatide [25].
- **Retatrutide (tri-agonist):** In a phase II trial (Eli Lilly, Indianapolis, IN, USA, 2023), retatrutide achieved an average weight reduction of 22% at 48 weeks—the largest ever recorded pharmacologically to date [30]. These results position retatrutide as a potential next-generation anti-obesity drug; it is in the final stages of study (phase III) and is not yet commercially available (experimental status). Cagrilintide + Semaglutide: a phase III study evaluating this combination reported 20,4% weight loss at 68 weeks—higher than semaglutide alone—with greater satiety and less rebound effect after termination [37]. This combined approach is being studied, indicating a future for multimodal peptide therapies for obesity.
- **Liraglutide, dulaglutide, and others:** Liraglutide (daily GLP-1 agonist) and dulaglutide (weekly) have also been shown to have cardiovascular benefits in high-risk diabetics. In the LEADER study, liraglutide reduced the rate of major adverse cardiovascular events by 13% compared with placebo [38]. Dulaglutide was associated with a 12% reduction in cardiovascular events in the REWIND study [39]. Weekly exenatide, in turn, had a neutral effect on mortality but still improved glycemic control and provided modest weight loss [40].
- **Dosage and Clinical Use:** Approved GLP-1 agonists are titrated to improve gastric tolerability. For example, semaglutide for obesity is initiated at 0.25 mg SC weekly and gradually increased to 2.4 mg/week [41]. Liraglutide is administered subcutaneously at 0.6 mg daily for the first week, then increased to 3.0 mg daily. Most common adverse effects include nausea, vomiting, and diarrhea at the beginning of treatment; usually manageable with slow titration. These peptides should not be used by individuals with a history of pancreatitis or medullary thyroid cancer [38,41].

#### 2.1.2. GH-Releasing Peptides: GHRH and GHRPs

- **GHRH Analogs:** These are peptides that mimic GHRH by directly stimulating GHRH receptors in the pituitary gland. Examples: drug affinity complex: growth hormone-releasing factor (CJC-1295); sermorelin; tesamorelin. They amplify pulsatile GH secretion, especially during sleep [16]. CJC-1295 stands out for containing a modification (DAC—drug affinity complex) that prolongs its half-life (~7–8 days), providing prolonged stimulation of the GH axis [10]. Tesamorelin, in turn, is a high-potency GHRH analog, approved for HIV-associated lipodystrophy because it reduces visceral fat [42].
- **GHRPs:** These include GHRP-2, GHRP-6, hexarelin, and ipamorelin. Unlike the ones mentioned previously, these peptides do not act on the GHRH receptor, but rather on the ghrelin receptor (GHS-R1a) in the pituitary gland and hypothalamus [10,15,42]. Activation of GHS-R1a triggers the phospholipase C (PLC) pathway, increasing intracellular calcium and releasing GH in an acute, pulsatile manner [15]. Each GHRP has its own profile: GHRP-6 and GHRP-2 are potent but increase prolactin and cortisol (especially GHRP-2); ipamorelin, on the other hand, is more selective, raising GH without elevating adrenocorticotropic hormone (ACTH)/cortisol [15,43]. Early studies showed that ipamorelin was the first GHRP not to stimulate cortisol significantly [15]. In short, GHRPs “mimic” endogenous ghrelin (hunger hormone) without fully reproducing its orexigenic effects—although some may slightly increase appetite [16].
- **Oral secretagogue (ghrelin mimetic):** MK-677 (Ibutamoren) is a non-peptide compound, but is often lumped together here because it acts as an oral GHS-R1a receptor agonist. It simulates the action of endogenous ghrelin by stimulating sustained GH and IGF-1 secretion for ~24 h with a single daily dose [5]. MK-677 has the advantage of an oral route and a long half-life and is being evaluated as a potential treatment for sarcopenia and growth disorders [23].
- **IGF-1 and Derivatives Growth Factors:** IGF-1 is the primary anabolic mediator of GH (produced in the liver and peripheral tissues). Synthetic derivatives include IGF-1 LR3 (Long R3 IGF-1, modified to not bind to carrier proteins, with extended half-life) and MGF (Mechano Growth Factor, an isoform of IGF-1 expressed locally in post-exercise muscle) [44,45,46]. These agents act directly on the IGF-1 receptor (a tyrosine kinase), strongly activating the PI3K-Akt-mTOR pathway, thereby promoting protein synthesis, muscle hypertrophy, and cell regeneration [16]. In theory, they could increase lean mass independently of GH secretion. However, the use of IGF-1 and analogs presents challenges, including hypoglycemia and potential pro-tumor effects, and warrants caution [44]. Indications and clinical approach: Analogs of GHRH and GHRPs have been evaluated for the treatment of GH axis disorders, including age-related GH deficiency and frailty syndromes (Table 2). Continued use can elevate IGF-1 and modestly increase lean mass and bone density, but improvements in physical performance are modest [16]. Tesamorelin has been shown to reduce visceral fat in patients with HIV [42], but outside of this context, its benefit is limited. MK-677 increased IGF-1 and appetite in older adults, resulting in a slight gain in muscle mass, but was associated with insulin resistance in some cases [5]. The use of IGF-1, LR3, and MGF is restricted to the experimental field or unauthorized use.
- **Safety, limitations, and regulatory status:** Lean mass and performance gains with these GH secretagogues are modest and are accompanied by metabolic (e.g., insulin resistance) and endocrine risks [16,23]. In addition, they are banned substances in sports, which restricts their use [10]. In summary, although they activate relevant anabolic pathways, the clinical cost–benefit of these peptides is still controversial, and their practical role is limited to very specific situations [16]. Future research may clarify whether physiological doses of these secretagogues could safely benefit elderly people with GH deficiency or patients with cachexia; for now, their use must be judicious [16,23].

#### 2.1.3. Regenerative and Tissue Repair Peptides

This category includes peptides that promote healing and tissue regeneration and modulate inflammation. It ranges from agents in the gastrointestinal tract and thymus to antimicrobial peptides, including BPC-157, Thymosin β4 (TB-500), Thymosin α1, and Copper glycylhistidyllysine (GHK-Cu), as well as emerging peptides such as Lys-Pro-Val tripeptide (KPV) and Cathelicidin antimicrobial peptide (LL-37) [55,56,57,58,59,60].

- **BPC-157 (Body Protection Compound-157):** It is a 15-amino acid peptide originally isolated from human gastric juice. It stood out for its stability in gastric acid for more than 24 h [27]. Its mechanism is multimodal: it stimulates angiogenesis (via increased VEGF and VEGFR2), modulates nitric oxide synthesis, accelerates granulation formation and collagen deposition, and reduces local inflammation [27,61]. Main targets: injured tissues—tendons, muscles, ligaments, bone, peripheral nerves, and gastrointestinal mucosa—according to preclinical evidence [11,62].
- **Thymosin β4 (TB-500)**: Thymosin β4 is a natural 43-amino acid protein produced by the thymus and other cells, with an important role in tissue repair. The synthetic fraction TB-500 corresponds to a key sequence (acetylated) of thymosin β4 responsible for its effects [63,64]. Mechanism: binds to monomeric actin (G-actin) in cells, promoting cell migration, progenitor cell recruitment, and angiogenesis [65]. It also exerts anti-inflammatory and anti-fibrotic effects, reducing aberrant collagen deposition [65]. The TB-500 fragment reproduces some of these effects, focusing on promoting new vessels and cell migration, and has even been used in veterinary medicine (e.g., doping in racehorses) [66].
- **Thymosin α1 (Thymosin α1):** Another peptide derived from the thymus, with 28 amino acids. Unlike TB-500, its primary effect is immunomodulatory: it activates toll-like receptor 9 (TLR9) and the NF-κB pathway in dendritic cells, thereby increasing the production of Th1 cytokines, interleukin-2 (IL-2), and Interferon-γ (IFN-γ), as well as the activity of cytotoxic T lymphocytes in restoring depressed cellular immunity, and is useful in immunosuppressed conditions [63,64]. It also has antiviral effects and has been used as a vaccine adjuvant. Clinical applications have included chronic hepatitis B and C (to improve antiviral response), certain cancers (to improve antitumor immune response), and, more recently, severe COVID-19 (to reduce sepsis and improve lymphopenia in critically ill patients) [67].
- **GHK-Cu**: It is a natural tripeptide (Gly-His-Lys) that binds strongly to the copper ion, forming the GHK-Cu complex. It mainly acts in dermal and connective tissue regeneration: GHK-Cu stimulates the production of collagen I and III, elastin, fibronectin, and glycosaminoglycans, in part by activating the growth factor TGF-β1 and the metalloproteinases (MMPs) [68]. It also has antioxidant and anti-inflammatory properties. Gene expression studies (e.g., the Broad Institute’s Connectivity Map) indicate that GHK-Cu can regulate the expression of up to 30% of human genes, thereby reversing the aging phenotype of cells to a more youthful pattern [68]. Targets and indications: improved healing (skin and cornea), regeneration of gastric ulcers, recovery of injured muscles and nerves, and skin anti-aging action [68,69].
- **KPV:** A short anti-inflammatory peptide derived from Alpha-melanocyte-stimulating hormone (α-MSH). Research primarily in preclinical models links it to gastrointestinal tract protection, promotion of mucosal healing, and enhanced epithelial wound repair. In intestinal epithelial cells and T cells, KPV is taken up via the PepT1 peptide transporter and suppresses NF-κB/MAPK signaling, thereby reducing pro-inflammatory cytokine levels [55,56]. Oral KPV reduces disease severity in colitis, with less histologic damage and lower cytokine expression, indicating improved mucosal integrity [57]. In a colitis-associated cancer model, KPV reduced tumor number and size when PepT1 was present, suggesting anti-inflammatory, barrier-protective effects that secondarily limit carcinogenesis [56].
- **LL-37**: The only human cathelicidin peptide. Beyond reducing microbial activity, it strongly influences epithelial barrier integrity, intestinal inflammation, and wound repair, making it a promising yet experimental therapeutic target. LL-37 is expressed in the gastrointestinal epithelium and contributes to the maintenance of colon mucosal barrier integrity and microbiota balance, with protective roles against colon tumorigenesis and colitis in animal models [58,59,60]. It enhances intestinal epithelial cell migration, induces protective mucins, and reduces apoptosis via P2X7 and EGFR signaling, helping re-establish barrier integrity after injury 10. Hybrid LL-37-based peptides (e.g., LL-37-TP5, LL-37–Tα1, Cecropin-LL37) reduce LPS- or EHEC-induced intestinal inflammation, restore tight junction proteins (ZO-1, occludin), and improve epithelial barrier function in mice [58,60,70].
- **Clinical applications:** BPC-157 has been used compassionately in sports medicine to accelerate recovery from musculoskeletal injuries, although it has not yet been approved by regulatory agencies [62]. Clinical trials are underway for conditions such as inflammatory bowel disease and orthopedic injuries. Thymosin β4 was evaluated in small case series of skin and eye lesions, demonstrating accelerated healing of pressure ulcers and corneal lesions [64]. Thymosin α1 is approved in some countries (e.g., Taiwan for hepatitis B) and is being evaluated for severe infections and cancer; in COVID-19, pilot studies have suggested reduced mortality among critically ill patients [68].
- **Safety:** Regenerative peptides have been well tolerated. BPC-157: no signs of significant toxicity have been reported in animals or in the limited human data; it does not appear mutagenic or immunogenic, and users have reported no consistent adverse effects, at most mild gastrointestinal distress at high oral doses [27]. Thymosin β4: in ulcer studies, there was no difference in adverse events vs. placebo; occasionally, itching or mild burning was observed at the site of topical application [71]. By taking a systemic approach, there have been rare reports of mild headaches. Thymosin α1: may cause transient redness at the injection site; no serious side effects have been reported in published studies [63,64]. GHK-Cu: topical is very safe (with very few cases of irritation); systemic is not yet used clinically, but animal studies have not indicated any relevant toxicity [68].

#### 2.1.4. Aesthetic and Dermatological Peptides (Cosmeceuticals)

These peptides primarily act on the skin and its appendages, targeting skin rejuvenation, improved texture, wrinkle reduction, and hair strengthening. They include GHK-Cu, several synthetic peptides used in cosmetics (such as the Matrixyl^®^ variants), and other palmitoylated tri/pentapeptides [68,69,72,73,74].

- **GHK-Cu:** As described previously, it is considered the “gold standard” of anti-aging peptides. It promotes the synthesis of collagen I and III, elastin, and other matrix components, stimulates angiogenesis, and acts as an antioxidant [68]. Its ability to modulate gene expression and reverse the aging phenotype in cells makes it a potent global skin rejuvenation agent [68]. Indications: improvement of wrinkles, firmness/sagging, scars, and even hair quality [69].
- **Palmitoyl Pentapeptide-4 (Matrixyl^®^):** Known by the KTTKS sequence attached to a palmitic fatty acid. It is a signaling peptide for fibroblasts: upon penetrating the dermis, it activates TGF-β and extracellular matrix signaling pathways, increasing collagen and fibronectin production [72]. One study has shown a reduction in fine wrinkles and lines with topical use over 2–4 months [73]. Because it is a small, lipophilic molecule (5 amino acids), it readily enters the skin. It is among the first and most studied cosmetic peptides, with proven efficacy in humans [73].
- **Matrixyl 3000:** A combination of two peptides (palmitoyl tripeptide-1 and palmitoyl tetrapeptide-7) that act synergistically by stimulating the extracellular matrix. In a 12-week clinical trial, Matrixyl 3000 demonstrated a significant reduction in wrinkles and lines, confirmed by silicone replication [69]. Increases in collagen I and fibrillin mRNA were also observed in treated skin [69].
- **Argirelin (Acetyl Hexapeptide-8):** A hexapeptide that mimics the N-terminal domain of SNAP-25, a component of the neurotransmitter release complex. In in vitro studies, concentrations of 0.005–0.05% of argylin inhibited 30–40% of neurotransmitter release at motor synapses, suggesting a “botox-like” effect [73]. Pure 10% Argireline has also been shown to significantly reduce forehead wrinkles when applied 2×/day for 30 days (measured by digital image analysis), with approximately 17–20% reduction in dynamic wrinkles [73]. No significant adverse effects, such as irritation, were observed—reinforcing its safety profile.
- **Common Dosage and Formulations:** These peptides are often used in cosmetics at relatively low concentrations but are effective when used repeatedly. Typical concentrations and average expected results at 8–12 weeks are summarized in Table 3.
- **Safety:** Cosmetic peptides have an excellent safety profile. They are non-toxic, non-irritating to most people, and have no measurable systemic effect (due to poor absorption beyond the dermis) [69]. Allergic reactions are very rare and are usually more related to the excipients in the formula than to the peptide itself. Thus, products containing these peptides can be used for extended periods as part of the skincare routine.

#### 2.1.5. Melanocortin Peptides (Sexual Function and Pigmentation)

Melanocortin peptides are synthetic analogs of the hormones alpha-MSH and ACTH that activate melanocortin receptors (MC1R–MC5R) in different tissues. They affect skin pigmentation processes, libido and sexual function, and even appetite and mood [73,74,75]. The main ones are: Afamelanotide (Melanotan I), Melanotan II, and Bremelanotide (PT-141). Melanocortin receptors and functions: MC receptors are distributed throughout the body: MC1R in melanocytes (controls melanin synthesis), MC3R and MC4R in the hypothalamus and limbic system (regulate eating behavior, sexual, and energy expenditure), and MC5R in exocrine glands and the central nervous system (secretory and metabolic functions) [73,74,75,76]. Thus, agonists at these receptors can produce diverse effects, ranging from skin tanning to increased libido and appetite suppression.

- **Afamelanotide (Melanotan-1):** Selective agonist of MC1R. It increases melanocyte eumelanin production, even without sun exposure, resulting in skin tanning and greater photoprotection (melanin absorbs UV radiation and prevents damage) [73]. It also has an anti-inflammatory effect on the skin. Main indication: erythropoietic protoporphyria (EPP), a rare disease in which patients have extreme photosensitivity. In patients with PPE, the administration of afamelanotide increased the duration of pain-free sun exposure and did not elicit neutralizing antibodies after multiple doses, demonstrating immunological safety [73]. Recent studies further suggest that afamelanotide may protect the skin from UV damage beyond tanning by increasing thymine dimer repair and reducing UV-induced reactive oxygen species [74].
- **Melanotan II:** Non-selective agonist of MC1R, MC3R, and MC4R. Originally developed as a tanning agent, a significant increase in libido and the occurrence of spontaneous erections in men have been observed as side effects. This led to the development of Bremelanotide (PT-141), a derivative specific for sexual function [75]. Melanotan II itself is used recreationally by some users to tan, but it is not medically approved due to adverse events such as nausea and increased blood pressure.
- **Bremelanotide (PT-141):** Mainly MC3R/MC4R agonist. Approved in the USA (2019) for the treatment of hypoactive sexual desire disorder (HSDD) in premenopausal women. Administered by SC injection on demand, about 30–45 min before intercourse. In studies, ~80% of men with erectile dysfunction had satisfactory erections with PT-141 vs. 30% with placebo, without causing significant hypotension even in nitrate users [75]. In women with HSDD, clinical trials have shown a significant increase in sexual desire and satisfaction with PT-141 compared to placebo [76]. Side effects may include nausea, facial flushing, and a mild increase in blood pressure; however, it is generally well-tolerated.
- **Setmelanotide:** MC4R agonist approved for monogenic obesity: Pro-opiomelanocortin (POMC), Proprotein convertase 1 (PCSK1), or leptin receptor deficiency. It reduces appetite, induces marked weight loss in these patients, and is associated with improved mood and energy [77,78]. In a study of individuals with congenital MC4R deficiency, setmelanotide led to significant weight loss and reported improvements in mood and vitality [78]. Adverse effects include nausea and darkening of the skin and hair. Setmelanotide exemplifies the principle of “pharmacogenomics”: targeted treatment for specific genetic mutations that cause obesity [77].
- **Safety:** Melanocortins may elevate blood pressure and cause nausea, especially Melanotan II. Afamelanotide (Melanotan-1) has been evaluated, with few adverse events other than hair darkening and freckling [73]. Bremelanotide can cause nausea in ~40% of users and mild headache, leading some to discontinue. It should not be used by patients with uncontrolled hypertension. Due to the potential for abuse (recreational use for aesthetic or sexual purposes), some of these unapproved peptides (Melanotan II) circulate illegally; its use is discouraged by risks and lack of quality control [79].

#### 2.1.6. Neuromodulator Peptides (Nootropics and Sleep)

They include peptides that affect the central nervous system, improving cognition, mood, and sleep, or providing neuroprotection. The main ones in this category are Semax, Selank, DSIP, and the analogous compound Cerebrolysin.

- **Semax:** A synthetic heptapeptide derived from a fragment of ACTH (Met-Glu-His-Phe-Pro-Gly-Pro), developed in Russia. It has significant nootropic and neuroprotective properties but lacks classic stimulant effects. Mechanism: potentiates the signaling of neurotrophic factors—increases the expression and release of Brain-derived neurotrophic factor (BDNF) in the hippocampus and cortex, leading to the activation of TrkB receptors [80]. It also modulates dopamine and serotonin levels in the brain and has a mild anti-inflammatory effect on the central nervous system [20]. Indications studied: sequelae of ischemic stroke, traumatic brain injury (TBI), mild cognitive disorders, depression, and Attention-Deficit/Hyperactivity Disorder [21]. In Russia, it has been used since the 1990s as an adjuvant in recovery from strokes and neurological injuries, and also in anxiety disorders and mild depression [19]. A Russian clinical study reported that intranasal Semax (0.1%) improved post-stroke neurological deficits and modulated inflammatory cytokines (IL-10, TNF-α) better than placebo [19]. In addition, in animal models, Semax accelerated motor recovery after spinal cord injury—an effect partially blocked by μ-opioid antagonist, indicating involvement of this pathway [18].
- **Selank:** A heptapeptide derived from a fragment of tuftysin (immunomodulatory peptide). It exhibits anxiolytic and nootropic properties without significant sedative effects. Mechanism: Selank is believed to modulate monoamine metabolism, increasing GABA release and regulating the expression of serotonin-related genes [81]. In parallel with Semax, it also elevates BDNF in certain areas (e.g., the prefrontal cortex) and exerts immunomodulatory effects (e.g., balancing Th1/Th2 responses). The result is a reduction in anxiety symptoms and an improvement in cognitive functions under stress, as observed in animal models and clinical reports [82]. In humans, intranasal Selank has been tested in patients with mild generalized anxiety disorder, leading to a significant reduction in anxiety scores after 2 weeks, with no sedative effects [82]. There are also data of improvement in symptoms of mild depression and mental fatigue [20].
- **DSIP (Delta Sleep-Inducing Peptide):** A natural 9-amino acid peptide found in the hypothalamus and cerebrospinal fluid. It was named for inducing slow-wave (delta) sleep in rabbits upon its discovery. Mechanism: probably acts at multiple sites; it is known to influence GABA and glutamate (NMDA) pathways [24]. The DSIP has a moderate hypnotic effect, promoting deep sleep and regulating circadian rhythms. In addition, it attenuates the stress response by reducing CRH and cortisol release in some contexts and increases GH pulses during sleep [15]. In studies, it exhibited anticonvulsant and neuroprotective effects. However, DSIP is rapidly degraded by specific peptidases in the cerebrospinal fluid (with a half-life of minutes), thereby hindering its therapeutic use [26].
- **Cerebrolysin:** Composed of a low-molecular-weight peptide mix (<10 kDa) derived from pig brain. It contains peptide fragments that mimic neurotrophic factors, including BDNF. When administered IV, these peptides can partially cross the blood–brain barrier and activate neuronal survival pathways (PI3K/Akt, MAPK) [83]. The result is neuroprotection, synaptogenesis, and improved post-injury plasticity. The drug has been used for decades in countries such as Austria, Russia, and China for recovery from stroke, TBI, and dementia, although it is viewed with skepticism by part of the Western medical community. Meta-analyses suggest modest but significant benefits for post-stroke motor recovery and cognitive function in patients with vascular dementia [83,84].
- **Clinical applications:** Semax and Selank are approved as drugs in Russia (Semax is officially indicated for stroke and neurological diseases; Selank for mild anxiety). In the rest of the world, they are not approved; they can be obtained from compounding pharmacies or the internet, but without registration with the main agencies [20]. WADA prohibits both, classifying them as peptides acting on the hormonal axis (category S2) or unapproved substances, for their potential neuroenhancement effect (even if there is no direct physical gain, it is considered cognitive doping) [85]. The DSIP was never officially approved; it circulates only as a research peptide, and WADA also prohibits it (S2 category) [85]. Cerebrolysin is approved in ~50 countries (primarily in Eastern Europe and Asia) for the treatment of neurological sequelae but has not been approved by the FDA [85]. Because it is used for medical treatment (e.g., stroke, dementia), it is not classified as a doping agent and would be permitted for athletes if prescribed for a medical condition (it does not qualify as a performance-enhancing agent) [83,84].
- **Safety:** Semax: It does not exhibit significant toxicity at the doses used; rare adverse events include mild nasal irritation (with intranasal administration) and transient agitation [19]. Selank: It is also considered safe, non-sedative, and non-addictive. Uncommon adverse effects include mild daytime drowsiness or dry mouth; it can be used for weeks to months with no reported complications [82]. DSIP: It is relatively safe, but because it induces sleep, it can cause excessive sleepiness or lethargy the next day if the dose is high. A mild drop in blood pressure (hypotension) has been reported during rapid IV infusion; in theory, prolonged use could suppress hormonal axes, although there is no clear evidence of this [24]. Cerebrolysin: The main adverse events reported are mild headache, nausea, feeling hot, and mild agitation in some patients, all of which are usually transient. Studies showed no increase in serious adverse events compared with placebo; attention should be paid only to the possibility of allergy in individuals sensitive to porcine proteins (the product’s raw material), although anaphylactic reactions are very rare [84].

#### 2.1.7. Myoanabolic Peptides (Myostatin–Follistatin Axis)

This class comprises peptide strategies to increase muscle mass by inhibiting myostatin or activating follistatin. Myostatin (GDF-8), a member of the TGF-β family produced by muscle cells, inhibits muscle growth; individuals or animals with myostatin deficiency exhibit pronounced muscle hypertrophy [86]. Thus, suppressing the myostatin pathway is a potential approach for treating sarcopenia and muscle diseases or for improving athletic muscle mass. Key peptides or proteins here include Follistatin (and variants) and soluble receptors like ACE-031/ACE-083, which act as myostatin “sponges.”

- **Follistatin:** Myostatin is secreted by muscles and circulates inactively bound to carrier proteins. When activated, it binds to activin receptor type 2 B (ACVR2B) on muscle fibers, triggering signaling pathways that inhibit protein synthesis and induce atrophy. Follistatin (FS), in turn, is an endogenous protein that binds to and neutralizes myostatin, activin, and other TGF-β ligands. Synthetic versions include Follistatin-344 (FS344, full isoform) and Follistatin-315 (circulating form) [87,88,89,90,91]. Follistatin is considered the most potent natural myostatin/activin antagonist; increasing its levels leads to overall muscle hypertrophy (as seen in myostatin knockout animal models) [88]. Therefore, experimental strategies, such as gene therapy and follistatin fusion proteins, have been explored to treat muscle diseases [87,89].
- **Soluble receptors (ACE-031, ACE-083):** These are fusion proteins that contain the extracellular domain of the ACVR2B receptor fused to IgG, acting as “bait” to bind circulating myostatin. ACE-031 was tested in children with Duchenne muscular dystrophy: despite increased lean mass and bone density, the phase II study was interrupted due to vascular adverse effects (telangiectasias, epistaxis), showing that broad systemic blockade of the TGF-β pathway requires caution [92]. More recent versions aim to restrict the action to the target muscle. ACE-083, for example, is applied directly to the desired muscle; in healthy volunteers and patients with neuromuscular diseases, intramuscular injections of ACE-083 increased local muscle volume by 30–50%, but without proportional functional improvement [93]. This suggests that gaining muscle alone is insufficient; it must be functionally integrated.
- **Experimental advances:** A milestone was the study by Kota et al. (2009) [89]: Rhesus monkeys received an AAV1 vector containing the FS344 gene in the leg muscles; this led to ~15% increase in local muscle mass without adverse systemic effects or changes in FSH levels (activin regulates FSH, and restriction of follistatin’s effect to muscle prevented reproductive impact) [89]. The animals maintained the gains for at least 1 year, demonstrating the durability of the effect [89]. In humans, a small study of patients with Becker muscular dystrophy evaluated intramuscular injections of AAV-follistatin and reported safety and modest strength gains, although no placebo control was included [94].
- **Effects on disease models and the elderly:** In elderly mice (24 months), delivery of follistatin via a viral vector increased muscle mass and strength, improved fiber size and neuromuscular junctions, and did not increase fat or fibrosis [88]. Improvement in muscle quality, not just quantity, is highlighted, possibly by normalizing trophic factors [88]. In older adults, there are no published clinical trials of follistatin; however, 8 weeks of resistance exercise reduces myostatin levels and increases endogenous follistatin and testosterone [90]. This suggests that, even without pharmacological intervention, exercise training favorably modulates this axis—and that a combined intervention (exercise + myostatin modulator) would be synergistic.
- **Safety:** In previous clinical trials, follistatin administration did not cause serious adverse events or alter hormones such as Follicle-Stimulating Hormone (FSH), demonstrating its specificity of action [89]. The adverse effects observed with ACE-031 (a systemic soluble receptor) reinforced the need to target therapy specifically to the muscle to avoid effects on other tissues [92]. All of these products are experimental; no myostatin modulator has been approved to date for sarcopenia or muscle gain in healthy humans. However, WADA has already banned any form of manipulation of the myostatin pathway—for example, the use of ACVR2B peptide or follistatin kits is considered doping and punishable [95]. In fact, products labeled as “follistatin peptide”, of dubious origin, have already circulated on the black market [91].

#### 2.1.8. Mitochondrial Peptides

Elamipretide (or SS-31) and MOTS-c (Mitochondria-Derived Open Reading Sequence of 12S rRNA type c) are therapeutic peptides that act on mitochondrial dysfunction, improving metabolic health and adenosine triphosphate (ATP) production [96,97,98,99]. MOTS-c is a mitochondria-encoded peptide that regulates metabolic homeostasis, while elamipretide is a synthetic tetrapeptide that stabilizes mitochondrial membranes, improving ATP production and reducing oxidative stress, particularly in muscles, including cardiac and skeletal muscle [96,97,98,99].

- **Elamipretide:** It is a tetrapeptide that selectively binds to cardiolipin in the inner mitochondrial membrane, stabilizing cristae, improving mitochondrial bioenergetics (ATP), and alleviating oxidative stress, without affecting normal mitochondria. Key indications include primary mitochondrial myopathy [99,100], Barth Syndrome [100], and heart failure with reduced ejection fraction [98].
- **MOTS-c:** It acts as a signaling molecule produced by mitochondria that, under increased demand (e.g., stress or exercise), can enter the nucleus, stimulating the regulation of genes related to metabolic flexibility and energy production [101]. Its action promotes glucose uptake, increases fatty acid metabolism, and reduces carbohydrate intake. Clinical studies show therapeutic potential for metabolic syndromes, obesity, and T2DM [101,102,103].
- **Clinical applications:** Elamipretide is already in phase 1–3 clinical trials and compassionate use, primarily for Barth syndrome, mitochondrial myopathies, and heart failure, with a favorable short-term safety profile and encouraging functional benefits [98,99,100,104]. MOTS-c, on the other hand, currently has no approved therapeutic application or is in late-stage development in humans, but extensive preclinical data support future clinical trials in metabolic diseases, cardiovascular complications, ischemia–reperfusion injuries, and chronic pain. Therefore, more data are needed to support its clinical indication [96,97,99,101,103].
- **Safety:** Elamipretide shows a generally good safety profile, with adverse effects mainly local (injection site reaction including: erythema, pruritus, pain, swelling, induration, and hematomas generally mild to moderate) and other less common symptoms such as headache, nausea, dizziness, and fatigue [98,99,100]; for MOTS-c, the data are almost exclusively preclinical, with no major signs of toxicity, but without robust evidence in humans [101,102,103]. Reviews of MOTS-c in T2DM emphasize its therapeutic potential but acknowledge the lack of human clinical trials to characterize safety, dosing, and adverse events [105].

## 3. Materials and Methods

The scientific literature was reviewed, and studies were retrieved from the PubMed (https://pubmed.ncbi.nlm.nih.gov/), ScienceDirect (https://sciencedirect.com/), and SciELO (https://scielo.br/) databases, accessed on 1 February 2026. Combinations of several search terms—such as “Therapeutic peptides”, “Injectable peptides”, “Regenerative peptides”, “athletic performance”, “GLP-1”, “BPC-157”, “elamipretide”, “tesamorelin”, “CJC-1295”, “MOTS-C”, “ipamorelin”, “TB-500”, “Semax”, “Selank”, “DSIP”, and “GHK-Cu”—were applied. After the search, studies were classified by health-specific parameters in the text and selected from 1982 to 2026, prioritizing systematic reviews, randomized controlled trials, and meta-analyses, resulting in a total of 106 articles. Our narrative review selected the effects and clinical applications of peptides as the primary outcome. Secondary outcomes include types and safety of therapeutic peptides in human health and sport performance. 

## 4. Discussion

Therapeutic peptides represent a growing class of drugs with applications in a variety of areas, including metabolic diseases, tissue regeneration, aesthetics, dermatology, oncology, and neurology. Its appeal lies in its high specificity, efficacy, and relatively favorable safety profile compared to traditional small molecules. However, challenges persist in immunogenicity, stability, impurity control, and regulatory compliance. Indeed, the accelerated development of peptides as incretin agonists (GLP-1/GIP), GH-releasing peptides (GHRH/GHRPs), melanocortins, neuromodulators, and mitochondrial peptides highlights the need for rigorous evaluations of their therapeutic effects, safety, and regulatory compliance.

As presented, therapeutic peptides offer substantial advantages in molecular specificity and a lower toxicological profile compared with conventional small molecules, especially in the metabolic/endocrine (GLP-1RAs) and dermatological/cosmetic areas. However, challenges persist in global regulatory standardization due to the structural and pharmacokinetic heterogeneity of these compounds, which hinder the universal definition of impurity limits and the development of robust predictive protocols for immunogenicity and tolerability. The paucity of robust randomized controlled trials in subclasses such as regenerative, neuromodulatory, and mitochondrial peptides limits definitive inferences about efficacy and safety in these emerging areas.

The increase in chemical strategies, such as cycling, has enabled the overcoming of classical limitations, such as proteolytic instability or low oral bioavailability, but has also introduced new potential risks associated with synthetic intermediates or by-products, which require close monitoring during clinical development and regulatory approval. Thus, this critical review synthesizes recent evidence on these aspects in different subclasses of therapeutic peptides.

## 5. Future Perspectives

With a greater understanding of therapeutic peptides, including new clinical research and innovative technologies such as artificial intelligence, the future of drug development can advance significantly, improving personalized treatment, reducing costs, and providing relief for diseases that do not respond to traditional treatments. Research on advanced stages with some peptides remains limited. However, the potential is enormous, as it enables exploration of the vast chemical space of peptide sequences and the identification of promising targets. This process can be accelerated through personalized patient analysis, including genomic and clinical data, and by studying peptides across various clinical conditions and applications.

Furthermore, the greatest challenge remains regulating the use of many of these peptides by industry and preventing misuse or incorrect dosing by recreational users. However, denying the therapeutic possibilities and stigmatizing therapeutic peptides as inappropriate therapies is a major mistake that should not be made by professionals with up-to-date knowledge and who understand the applications and safety of using these substances.

## 6. Conclusions

The current landscape of peptide therapy is rapidly expanding as a therapeutic option for numerous conditions. Established applications include GLP-1 agonists, which are revolutionizing the treatment of T2DM and obesity, and recently approved peptides like tesamorelin and elamipretide. Similarly, experimental treatments include effective cosmetic peptides for skin rejuvenation and rare hormone analogs under development for specific applications. Other peptides are in early stages of research, so their safety in humans remains uncertain. Healthcare professionals must ensure their knowledge and practice remain up to date on peptides and critically evaluate current evidence and its limitations for human application. While peptide therapy is a strategic and innovative option that can improve health, performance, and longevity, more studies are needed before most peptides can be safely used in humans.

## Figures and Tables

**Table 1:** Nomenclature, molecular structure, and regulatory status of therapeutic peptides.

Peptide	Molecular Structure	Regulatory Status (Year)	Additional Information
Liraglutide	C_172_H_265_N_43_O_51_	FDA-approved (2010)	* The 503B Bulk Drug Substances List (or 503B Bulks List) is an FDA-maintained list of active pharmaceutical ingredients that outsourcing facilities can use for compounding when there is a clinical need. Drugs compounded using these substances can bypass the requirement to use an approved drug as the starting material.
Dulaglutide	C_2646_H_4044_N_704_O_836_S_18_	FDA-approved (2014)	
Semaglutide	C_187_H_291_N_45_O_59_	FDA-approved (2017)	
Tirzepatide	C_225_N_348_N_48_O_68_	FDA-approved (2022)	
Retatrutide	C_221_H_342_N_46_O_68_	not yet FDA-approved	
Cagrilintide	C_194_H_312_N_54_O_59_S_2_	not yet FDA-approved	
Setmelanotide	C_49_H_68_N_18_O_9_S_2_	FDA-approved (2020)	
Tesamorelin	C_221_H_366_N_72_O_6_7_S_	FDA-approved (2010)	
Ipamorelin	C_38_H_49_N_9_O_5_	FDA 503B * Bulks List (2023)	
CJC-1295	C_165_H_269_N_47_O_46_	not yet FDA-approved	** The 503A bulks list is an FDA-established list of bulk drug substances that, while not approved by the FDA, are approved for use by licensed pharmacists in traditional pharmacy compounding under Section 503A of the Federal Food, Drug, and Cosmetic Act.
Sermorelin	C_149_H_246_N_44_O_42_S	FDA 503B * Bulks List (2020)	
Hexarelin	C_47_H_58_N_12_O_6_	not yet FDA-approved	
MK-677 (Ibutamoren)	C_27_H_36_N_4_O_5_S	FDA 503A ** Bulks List (2023)	
IGF-1 LR3	C_400_H_625_N_111_O_115_S_9_	not yet FDA-approved	
GHRP-6	C_46_H_56_N_12_O_6_	FDA 503B * Bulks List (2023)	
GHRP-2	C_45_H_55_N_9_O_6_	FDA 503B * Bulks List (2023)	
BPC-157	C_62_H_98_N_16_O_22_	FDA 503A ** Bulks List (2023)	
Thymosin β4	C_212_H_350_N_56_O_78_S	FDA 503A ** Bulks List (2023)	
GHK-Cu	C_14_H_24_N_6_O_4_Cu_1_	FDA 503A ** Bulks List (2023)	
KPV	C_16_H_30_N_4_O_4_	FDA 503A ** Bulks List (2023)	
LL-37	C_205_H_340_N_60_O_53_	FDA 503A ** Bulks List (2023)	
Palmitoyl Pentapeptide-4	C_39_H_75_N_7_O_10_	not yet FDA-approved	
Matrixyl 3000	mixture of peptides	not yet FDA-approved	
Argirelin	C_34_H_60_N_14_O_12_S	not yet FDA-approved	
Afamelanotide	C_78_H_111_N_21_O_19_	FDA-approved (2019)	
Melatonan II	C_50_H_69_N_15_O	FDA 503A ** Bulks List (2023)	
Bremelanotide	C_50_H_68_N_14_O_10_	FDA-approved (2019)	
Semax	C_37_H_51_N9O_10_S	FDA 503A ** Bulks List (2023)	
Selank	C_33_H_57_N_11_O_9_	not yet FDA-approved	
DSIP	C_35_H_48_N_10_O_15_	not yet FDA-approved	
Cerebrolysin	mixture of peptides	not yet FDA-approved	
Follistatin-344	C_1640_H_2520_N_428_O_496_	not yet FDA-approved	
ACE-031	C_133_H_227_N_43_O_33_	not yet FDA-approved	
MOTS-C	C_101_H_152_N_28_O_22_S_2_	FDA 503A ** Bulks List (2023)	
Elamipretide	C_32_H_49_N_9_O	FDA-approved (2025)	

Abbreviation: ACE: Myostatin inhibitory peptide; BPC-157: Body Protection Compound 157; CJC-1295: drug affinity complex: growth hormone-releasing factor; DSIP: delta sleep-inducing peptide; FDA: Food and Drug Administration; GHK-Cu: Copper glycylhistidyllysine; GHRP: growth hormone-releasing peptides; IGF-1: insulin growth factor 1; KPV: Lys-Pro-Val tripeptide; LL-37: cathelicidin antimicrobial peptide; MK-677: Ibutamoren; MOTS-c: mitochondrial open reading frame of the 12S rRNA-c.

**Table 2:** Doses, advantages, and side effects for GH-releasing peptides and analogs.

Peptide	Dose and Route	Advantages	Side Effects	References
Tesamorelin	2 mg SC daily	Increases GH/IGF-1; reduces visceral adiposity in HIV patients (FDA-approved)	Injection site reactions; mild edema	[47]
Ipamorelin	0.03–1.0 μg/kg IV or SC	Selective GH release; minimal effect on prolactin/cortisol	Well-tolerated; rare flushing	[22,48]
CJC-1295	30–60 μg/kg SC weekly	Long-acting GHRH analog; sustained GH/IGF-1 elevation	Mild injection site reactions	[48,49]
Sermorelin	0.2–0.3 mg SC daily	Mimics endogenous GHRH; stimulates pituitary GH release	Flushing; injection site discomfort	[47,48]
Hexarelin	60 μg/kg intranasal TID (children) 0.1–1 μg/kg IV/SC (adults)	Strongest peptide for GH release; increases IGF-1	Transient cortisol and prolactin rise	[47,50,51]
MK-677 (Ibutamoren)	10–25 mg oral daily	Orally active; increases GH/IGF-1 and lean mass	Increased appetite; mild edema	[47,48,52]
IGF-1 LR3	20–100 μg/kg SC daily	Direct IGF-1 action: anabolic effects	Hypoglycemia risk	[48]
GHRP-6	0.1–1 μg/kg IV/SC/oral	Potent GH release; oral activity (low bioavailability)	Mild flushing; increased appetite	[22,53]
GHRP-2	0.3–3 μg/kg SC or oral	Potent GH secretagogue; increases appetite	Transient hunger; well-tolerated	[47,54]

Abbreviation: FDA: Food and Drug Administration; GH: growth hormone; GHRP: growth hormone-releasing peptides; HIV: human immunodeficiency virus; IGF-1: insulin growth factor 1; IV: intravenous; SC: subcutaneous; TID: three times a day.

**Table 3:** Typical concentration and average result of dermatological peptides.

Peptide	Concentration	Formulation	Average Results (8–12 Weeks)
GHK-Cu	0.01–0.05%	Serum or cream	~30% wrinkle reduction; +30–35% Firmness
Palmitoyl Pentapeptide-4 (Matrixyl)	0.03–0.1%	Cream/Gel	~20–30% wrinkle reduction
Matrixyl 3000	0.05–0.2%	Lotion/serum	~33% wrinkle reduction
Argirelina (Acetyl Hexapeptide-8)	0.005–0.1%	Light serum/cream	~25–30% reduction in dynamic wrinkles
Novel Peptides (Pal-GHK-R4)	~0.01–0.05%	Nanoemulsion	+20% hydration; ~30% wrinkle reduction

Abbreviation: GHK-cu: Copper glycylhistidyllysine.

## Data Availability

No new data were created or analyzed in this study. Data sharing is not applicable to this article.
